# Dual immune armies in glaucoma: microglia and monocyte-derived macrophages

**DOI:** 10.3389/fimmu.2026.1801497

**Published:** 2026-04-02

**Authors:** Kang Tan, Yan Zhang, Pengfei Jiang, Yunfeng Yu, Zizheng Wu, Jun Peng, Pei Liu, Qinghua Peng

**Affiliations:** 1 Hunan University of Chinese Medicine, Changsha, China; 2 The First Hospital of Hunan University of Chinese Medicine, Changsha, China; 3 Quzhou Hospital Ophthalmology Center, Zhejiang Medical and Health University, Quzhou, China

**Keywords:** cell polarization, glaucoma, inflammation, microglia, monocyte-derived macrophages

## Abstract

Glaucoma is a leading cause of irreversible blindness globally, with the core pathological feature being progressive degeneration of retinal ganglion cells. Neuroinflammation pervades the entire course of glaucoma, and an imbalance in the intraocular immune microenvironment is critical factor underlying progression. As an extension of the central nervous system, the retina has a unique immune microenvironment. Under physiological conditions, microglia, which are primary tissue-resident immune cells, maintain homeostasis. In pathological states, the blood-retinal barrier is compromised and allows monocyte-derived macrophages (MDMs) to infiltrate the retinal tissue. We introduce the concept of “dual immune armies, ” specifically referring to the core retinal immune population comprising microglia and MDMs. These two cell types coordinate to form an immune network, but they demonstrate significant functional heterogeneity during glaucoma pathogenesis. Microglia act as first responders and activate rapidly during the early stages of injury, monitor changes in the microenvironment in real time, and initiate the primary inflammatory response. MDMs serve as “late-reinforcement troops” and infiltrate extensively following blood-retinal barrier disruption, amplify the inflammatory cascade, and exacerbate optic nerve damage. Previous studies have often conflated these two cell types, leading to a lack of precise targets for immune intervention in glaucoma. Based on recent research, this study systematically compared the origins, functions, and specific marker profiles of microglia and MDMs with a focus on elucidating their synergistic roles and functional division of labor in glaucomatous optic neuropathy. Elucidating the heterogeneity of these two immune cell populations and their precisely regulated functions at different disease stages will help clarify the key mechanisms underlying the imbalance of the retinal immune microenvironment in glaucoma. It will also provide a new theoretical basis and research direction for the development of targeted immunomodulatory strategies to protect retinal ganglion cells and potentially reverse optic nerve damage.

## 1 Introduction

In recent years, research on neuroimmune regulation has advanced substantially in multiple central nervous system (CNS) neurodegenerative disorders, including Alzheimer’s disease, Parkinson’s disease, and amyotrophic lateral sclerosis (1–4). Accumulating evidence indicates that endogenous and peripherally derived immune cells jointly constitute a complex immunoregulatory network that plays pivotal roles in neuroprotection and tissue repair (5). As an extension of the CNS, the retina is governed by a neuroimmune network (6). Neuroinflammation is persistent throughout the course of glaucoma and has emerged as a central pathogenic mechanism driving the progressive loss of retinal ganglion cells (RGCs) (7).

The onset and progression of glaucoma is accompanied by profound remodeling of the retinal immune microenvironment (8, 9). Under physiological conditions, the blood-retinal barrier (BRB) effectively restricts the infiltration of peripheral immune cells, whereas yolk sac-derived microglia, the principal innate immune sentinels, maintain tissue homeostasis through continuous surveillance and synaptic remodeling (10, 11). However, under pathological insults such as elevated intraocular pressure (IOP), ischemia/hypoxia, or oxidative stress, injured RGCs release large amounts of danger-associated molecular patterns (DAMPs) and pro-inflammatory cytokines (such as TNF-α and IL-1β), rapidly disrupting retinal immune privilege (12). These injury signals not only directly activate microglia but also compromise BRB integrity (13). Concurrent with robust chemokine release (such as C-C motif chemokine ligand 2 [CCL2]), peripheral monocytes traverse the damaged BRB, infiltrate the retina and optic nerve region, and differentiate into monocyte-derived macrophages (MDMs) (14). Consequently, microglia and MDMs together form “dual immune armies” that drive glaucomatous neuroinflammation, and their interactions critically influence the outcome of optic nerve injury. During the early stage of the disease, microglia act as first responders, exerting predominantly protective functions such as debris clearance and secretion of neurotrophic factors (15). With disease progression, the markedly increased influx of MDMs is characterized by stronger phagocytic activity and a greater propensity for pro-inflammatory polarization. Through paracrine signaling, MDMs release cytotoxic mediators that further activate microglia, exacerbate local microenvironmental deterioration, and shift the balance from neuroprotection toward neurotoxicity (16). In parallel, these two macrophage populations engage in complex molecular crosstalk with astrocytes and Müller glia, inducing reactive gliosis and ultimately establishing a self-amplifying, multicellular immune cascade (17). This dynamic transition from compensatory cooperation during the early stages to pathological imbalance during the later stages for microglia and MDMs represents a key driver of progressive RGC death (18).

The central roles of microglia and MDMs in glaucomatous neuroinflammation are widely acknowledged, but prior studies have often conflated these populations (19, 20). Their morphologies and expressions of conventional markers (such as Iba1, CD68, and F4/80) are similar, and they have frequently been collectively described as a “macrophage/microglia complex” (21, 22). This conceptual ambiguity, together with methodological limitations, has obscured the stage-dependent functional differences between the two cell types and may partly explain why non-selective immunomodulatory interventions targeting pan-macrophage pathways have limited efficacy in preclinical studies, thereby hindering precise neuroprotective strategies. Recent rapid advances in single-cell RNA sequencing (scRNA-seq), spatial transcriptomics, and cell type-specific genetic lineage-tracing approaches have enabled the accurate discrimination of these populations at the transcriptomic and epigenetic levels (23–25). Emerging evidence demonstrates that microglia (characteristically expressing TMEM119 and P2RY12) and MDMs (characteristically expressing CC chemokine receptor 2 [CCR2] and Lymphocyte antigen 6C [Ly6C]) are highly heterogeneous populations that have substantially different disease signal responsiveness, polarization trajectories, metabolic reprogramming, and spatiotemporal distribution (26).

Accordingly, this review synthesizes the latest progress on “dual immune armies” in glaucoma. We systematically compared microglia and MDMs in terms of their developmental origins, specific markers, phenotypic transitions, and functional contributions to glaucomatous optic nerve injury. We further highlight their immune interactions and dynamic evolution across disease stages. Clarifying the respective cooperation and division of labor between these two populations will not only deepen the mechanistic understanding of glaucomatous neuroinflammation but also provide a theoretical basis for developing precision interventions that selectively target specific immune cell subsets.

## 2 Microglia and MDMs in the retina

### 2.1 Microglia

**Figure 1:**
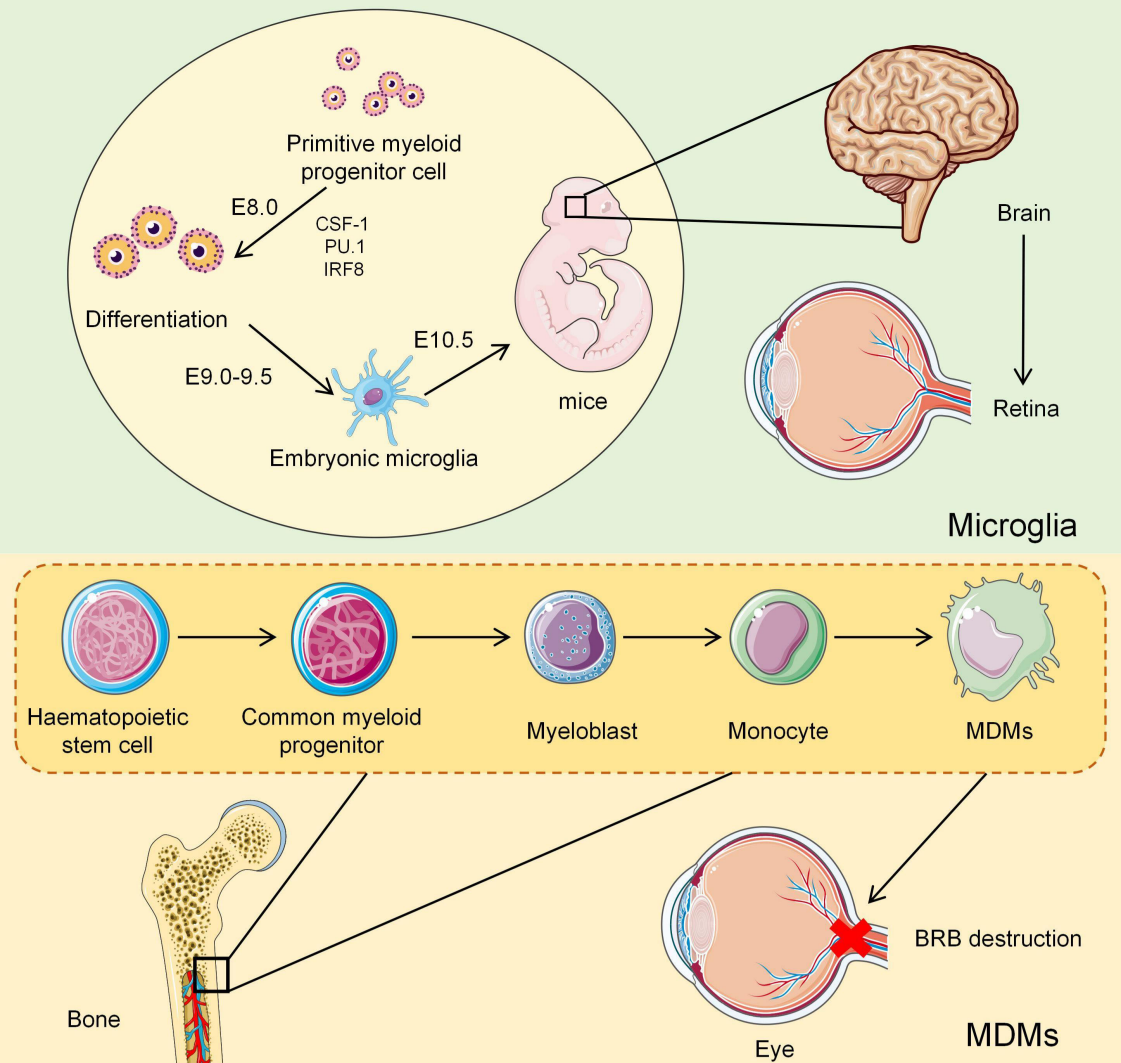
Schematic diagram of developmental origins and colonization pathways of retinal microglia and MDMs. Microglia originate from primitive myeloid progenitor cells in the embryonic yolk sac. Around E8, these progenitor cells initiate directed differentiation under the synergistic regulation of CSF-1 signaling and key transcription factors PU.1 and IRF8. They form early glial cells during the E9.0-E9.5 stage, migrate into the developing CNS at E10.5, and ultimately settle in the retina. In contrast, MDMs originate from adult bone marrow hematopoietic stem cells and develop through the peripheral blood monocyte stage. Under steady-state conditions, circulating monocytes rarely enter ocular tissues. However, they are recruited, traverse the BRB, infiltrate the retina, and participate in the local immune response during retinal inflammation, injury, or pathological changes.

Retinal microglia represent the ocular extension of the CNS microglia and have a distinct developmental origin (27). They originate from primitive myeloid progenitor cells in the embryonic yolk sac (28). During murine embryogenesis, these progenitors are regulated by colony-stimulating factor 1 and rely on the transcription factors PU.1 and IRF8 for signal transduction, initiating lineage-specific differentiation on embryonic day 8 (29, 30). By embryonic day 9.0–9.5, immature microglia are formed (31). On day 10.5, they migrate to the developing brain and colonize the retina (**Figure 1**) (32). The exact migratory route to the retina remains controversial; some studies have suggested entry via the optic nerve head (33), whereas others support migration through the ciliary body (34, 35). After retinal colonization, microglia have stratified distribution (36). They are most abundant within the synaptic plexiform layers of the inner retina, where they arranged in horizontal arrays. On the other hand, they are sparsely distributed in the outer retina (31). Under pathological conditions or aging, microglia may relocate to the outer retinal layers and subretinal space (37). In their resting state, microglia have a highly ramified morphology characterized by small somata, sparse cytoplasm, and long, motile processes engaged in active surveillance (38). Upon activation, they rapidly transition to the amoeboid form (39).

**Figure 2:**
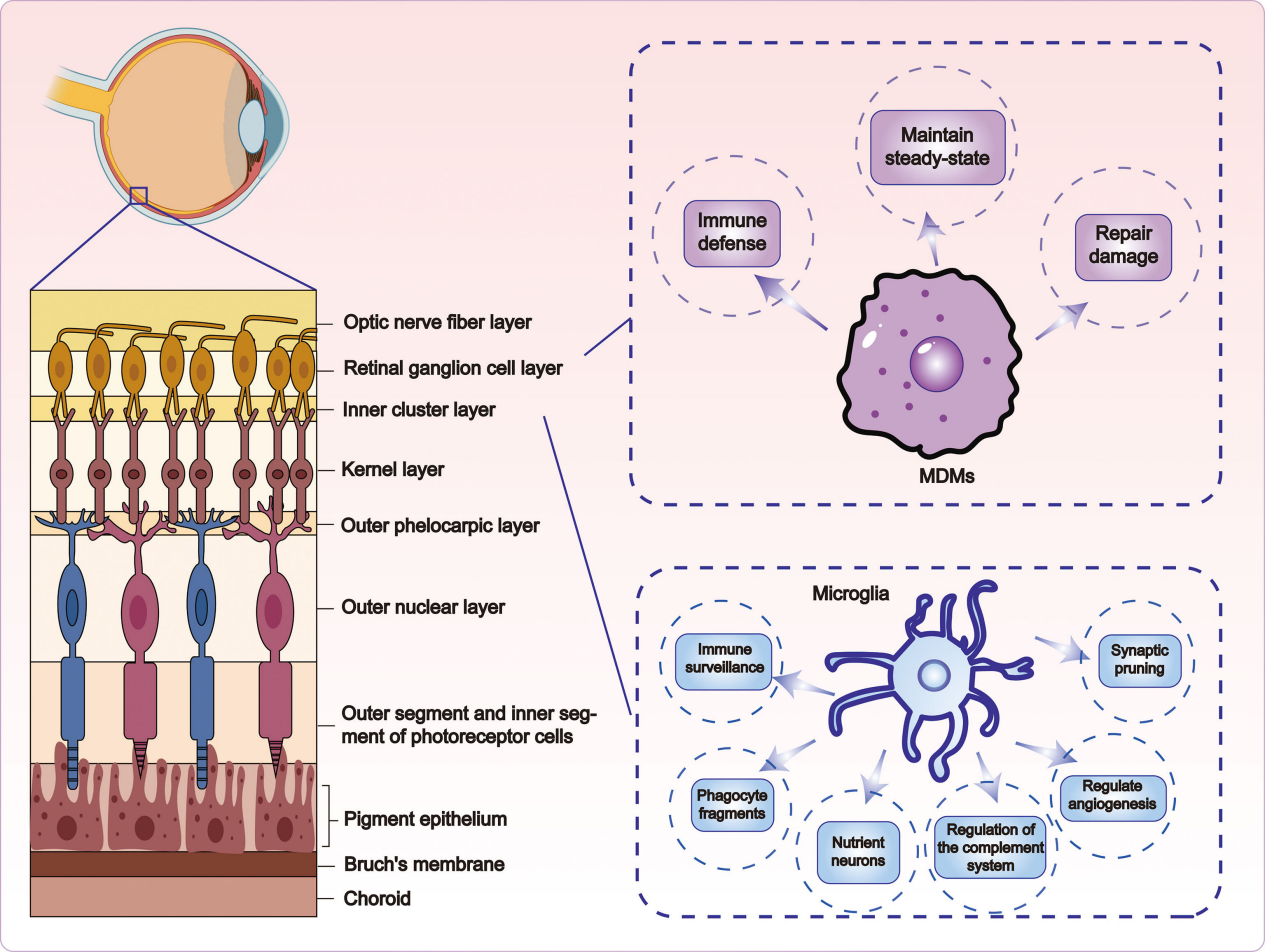
Functions of microglia and MDMs. Microglia perform essential functions including immune surveillance, synaptic pruning, clearance of apoptotic cells and cellular debris, regulation of vascular development, secretion of neurotrophic factors such as brain-derived neurotrophic factor (BDNF), and modulation of complement activity (e.g., C1q, C3, and C5), whereas MDMs primarily contribute to immune defense, tissue homeostasis, and repair.

Microglia in the adult retina are long-lived cells (40) capable of maintaining homeostasis by clearing damaged organelles via autophagy (41). Under physiological conditions, these cells self-renew through slow local proliferation without requiring peripheral input, whereas they undergo rapid clonal expansion in diseased states (42). Their core functions are diverse (**Figure 2**). First, they serve as primary immune sentinels, continuously surveying the microenvironment through dynamic extension and retraction of their processes to detect subtle perturbations (18). Second, synaptic pruning is actively mediated during early neural circuit development and remodeling to refine and optimize neuronal connectivity (43). Third, they act as key phagocytes and clear apoptotic cells and metabolic debris to preserve retinal homeostasis (44). Fourth, microglia modulate vascular development by participating in retinal angiogenesis and vessel maturation (45). Fifth, they secrete neurotrophic factors—such as brain-derived neurotrophic factor (BDNF)—which provide trophic and protective support to neurons (46). Sixth, they regulate the complement system through the synthesis and secretion of complement components (such as C1q, C3, and C5) and fine-tune local immune responses (47). Moreover, microglia form intricate interaction networks with other retinal glial cells, including Müller cells and astrocytes, and collectively maintain a dynamic equilibrium of the retinal microenvironment (48).

### 2.2 MDMs

MDMs originate from hematopoietic stem cells in the adult bone marrow and differentiate into circulating monocyte subsets (49). Under physiological conditions, peripheral monocytes rarely infiltrate intraocular tissues (50). In the presence of retinal inflammation, injury, or other pathological changes, they are recruited and traverse the BRB into the eye (**Figure 1**) (51). In experimental models of glaucoma, MDMs frequently infiltrate injury-associated regions, including the retinal ganglion cell layer, optic disc, and choroid (52). Morphologically, they typically appear rounded or amoeboid and have markedly reduced or absent branching processes (53).

Unlike microglia, MDMs lack self-renewal capabilities and rely entirely on continuous recruitment from circulating monocytes (54). After the resolution of inflammation or tissue repair, a subset may integrate into the microglial network but does not fully differentiate into true microglia, retaining distinct transcriptional and functional characteristics (55). Resident MDMs have altered functions and may display diminished responsiveness to subsequent injuries (51). Functionally, MDMs are primarily involved in immune defense, tissue homeostasis, and injury repair (**Figure 2**) (56). Upon inflammation or damage, they are rapidly recruited to lesion sites via chemokines such as C-C motif chemokine ligand 2 (CCL2) and C-X3-C motif chemokine ligand 1 (CX3CL1) (57). They clear pathogens and necrotic cells through phagocytosis, present antigens, and secrete pro-inflammatory mediators including TNF-α and Interleukin-1β (IL-1β) (58).

Retinal microglia and MDMs share phagocytic capacity and have a degree of phenotypic plasticity, allowing them to adapt their functional states to local microenvironmental signals. Therefore, they constitute the core innate immune surveillance system of the retina. However, they fundamentally differ in their origins and maintenance mechanisms. Microglia are embryonically established, lifelong self-renewing “resident custodians that are intimately integrated into the retinal microenvironment and are primarily responsible for maintaining neural tissue homeostasis. In contrast, MDMs are bone marrow-origin “mobile sentinels” dependent on peripheral replenishment. Extensive infiltration often indicates BRB disruption or homeostatic imbalance, and their primary role is to counter acute injury and clear pathogens. These differences in developmental origin and population maintenance profoundly shape responsiveness, functional output, and long-term impact on retinal microenvironmental changes.

## 3 Markers of microglia and MDMs

### 3.1 Shared markers

**Figure 3:**
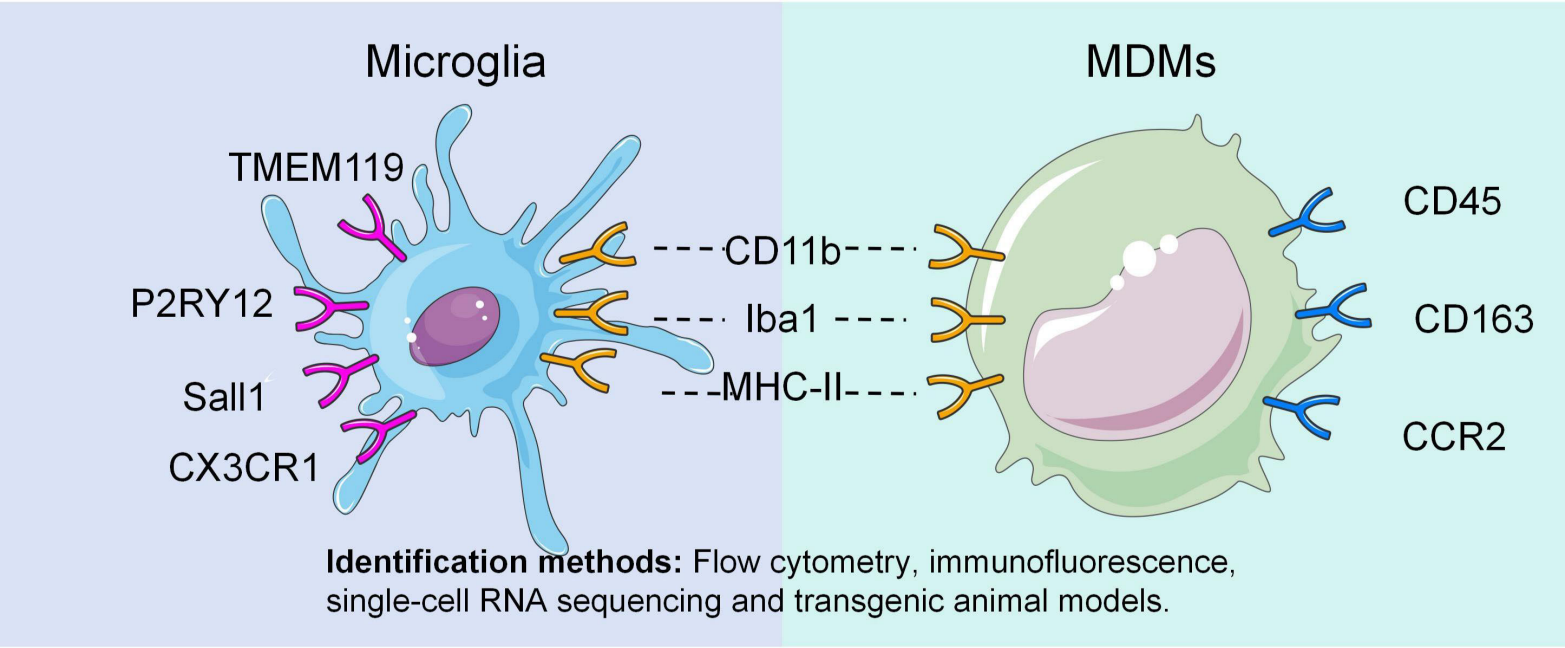
Cellular markers of microglia and MDMs.

As integral components of the innate immune system, microglia and MDMs share a set of fundamental immunophenotypic markers primarily associated with phagocytic capacity and antigen-presenting ability. The expression of these markers is typically upregulated upon cell activation, making them reliable indicators of immune response (**Figure 3**).

CD11b (integrin αM subunit) is part of complement receptor 3 (CR3) (59) and one of the most widely recognized surface markers for myeloid cells, including microglia and MDMs (60, 61). In laser-induced chronic ocular hypertension models, CD11b expression in microglia is markedly increased, which correlates with morphological transformation, migratory behavior, and enhanced phagocytosis (62). Given its consistent and robust expression in the activated state, CD11b is frequently used as a primary marker for identifying these cell populations (63, 64).

Ionized calcium-binding adaptor protein 1 (Iba1) is a cytoplasmic protein highly expressed in microglia and MDMs and plays a key role in cytoskeletal reorganization and phagocytosis (65). Elevated Iba1 expression levels in postmortem retinal specimens from patients with glaucoma have been documented as reliable indicators of microglial and MDMs activation (66). Due to its robust staining properties, Iba1 antibodies are widely used to locate these cells in tissue sections and assess morphological changes (67).

Major histocompatibility complex class II (MHC-II) is essential for antigen presentation (68). Microglia express MHC-II at low levels in a resting state. In the presence of pathological stimuli such as glaucoma, however, both activated microglia and infiltrating MDMs have substantially elevated MHC-II expressions (69).

These shared markers highlight the overlapping core functions of both cell types in phagocytosis and the initiation of immune responses. Nevertheless, relying solely on these markers is insufficient for precise discrimination under inflammatory conditions and necessitates the use of more specific identifiers.

### 3.2 Microglia-specific markers

As resident macrophages in the CNS, microglia have a distinct gene expression profile shaped by long-term evolution that distinguishes them from MDMs (**Figure 3**).

Transmembrane protein 119 (TMEM119), a type I transmembrane protein, is widely regarded as a specific microglial marker (70). TMEM119 is stably expressed in microglia under both homeostatic and activated conditions but is almost absent in MDMs (71). Consequently, it is often considered the “gold standard” for distinguishing retinal microglia from MDMs.

The purinergic receptor P2Y12 is another highly specific marker that mediates the chemotactic response of microglia to extracellular ATP gradients, which is a hallmark of tissue injury signaling (72). Studies have shown that apoptotic cell clearance in the retina is synchronized with microglial phagocytic activity and depends on P2RY12 signaling; blocking this pathway delays the removal of apoptotic cells (73).

Sall1, a transcription factor critical for microglial development and homeostasis, is a highly specific and stable marker. Notably, its expression was maintained even during severe activation (74).

CX3C chemokine receptor 1 (CX3CR1), the receptor for fractalkine (CX3CL1), is a G protein-coupled receptor expressed by both microglia and MDMs, but with distinct patterns. Microglia express high levels of CX3CR1, and its signaling is closely linked to cell proliferation, activation, and neurovascular unit function (45, 75). In MDMs, CX3CR1 expression is low or dynamically regulated, primarily facilitating cell migration without modulating pro-inflammatory cytokine secretion (76). Therefore, high CX3CR1 expression is often used as an auxiliary marker to identify microglia.

### 3.3 MDMs-specific markers

Recruitment of MDMs from circulation into the retina confers features typical of the peripheral immune system, with markers largely associated with cell trafficking, recognition, and an adaptable inflammatory state (**Figure 3**).

Both cell types express CD45, but the levels differ markedly; the level ofCD45 expression is low for microglia and high for MDMs (77). Ly6C is a marker of inflammatory monocytes. MDMs are typically Ly6C^+^, whereas microglia are Ly6C^-^ (78). Therefore, identifying the combination of CD45 and Ly6C expressions enables precise identification of MDMs.

CD163, a scavenger receptor, reflects M2-type MDM polarization (79). Immunohistochemical comparison of normal and glaucomatous optic nerves has revealed increased numbers of CD163^+^ cells in both mild and severe glaucoma, confirming MDM infiltration in glaucomatous optic nerve tissue (80).

CCR2, a G−protein−coupled receptor for CCL2, mediates MDM migration toward inflammatory sites (81) and serves as a robust identifier for MDMs. Elevated levels of expression of CCL2, CXCR2, and CCR2 have been linked to increased aqueous humor production and the pathogenesis of primary open-angle glaucoma (82).

### 3.4 Strategies for differentiating markers of microglia and MDMs

The accurate differentiation of microglia from MDMs is essential for elucidating neuroinflammatory processes, RGC injury, and optic nerve degeneration. Current approaches involve integrating multiple cell biology techniques and marker combinations as mainstream strategies for discrimination.

Flow cytometry is the gold standard for quantitative analysis and sorting. A typical workflow involves gating myeloid cells with CD11b^+^, followed by stratification based on CD45 expression (CD45^low^ for microglia, CD45^high^ for MDMs) and final confirmation using Ly6C (Ly6C^-^ for microglia, Ly6C^+^ for MDMs) (83).

Immunofluorescent double- or multiplex-labeling of retinal sections allows direct visualization of spatial distribution, morphological changes, and activation states. Broad-spectrum myeloid markers (such as Iba1) are often combined with cell-type-specific markers (such as TMEM119 for microglia), with a focus on crucial regions such as the ganglion cell layer under glaucomatous conditions (84).

Single-cell RNA sequencing (scRNA-seq) or bulk transcriptomic analysis enables the comprehensive profiling of gene expression differences between these cell populations. Comparative transcriptomics for glaucoma models can identify functional states, activated signaling pathways, and subpopulation heterogeneity and provide molecular-level insights into their distinct roles in disease progression (85).

Lineage tracing and cell-specific labeling in transgenic models enable *in vivo* visualization and functional manipulation of targeted cell populations. For example, CX3CR1-CreER mice can be used to label and track the lineage and dynamics of retinal microglia (86), whereas CCR2-RFP mice mark peripheral CCR2^+^ MDMs (87). In combination with ocular hypertension or optic nerve injury models, these tools allow the real-time observation of the spatiotemporal behavior and interactions of both cell types during glaucoma progression.

Phenotypic profiles may shift under chronic neuroinflammatory conditions. Evidence suggests that MDMs with prolonged CNS residence express microglia-specific markers (26). Therefore, caution is advised when defining and applying such specific markers, and multi-marker and multi-technique approaches are recommended for accurate discrimination.

## 4 Polarization of microglia and MDMs

**Figure 4:**
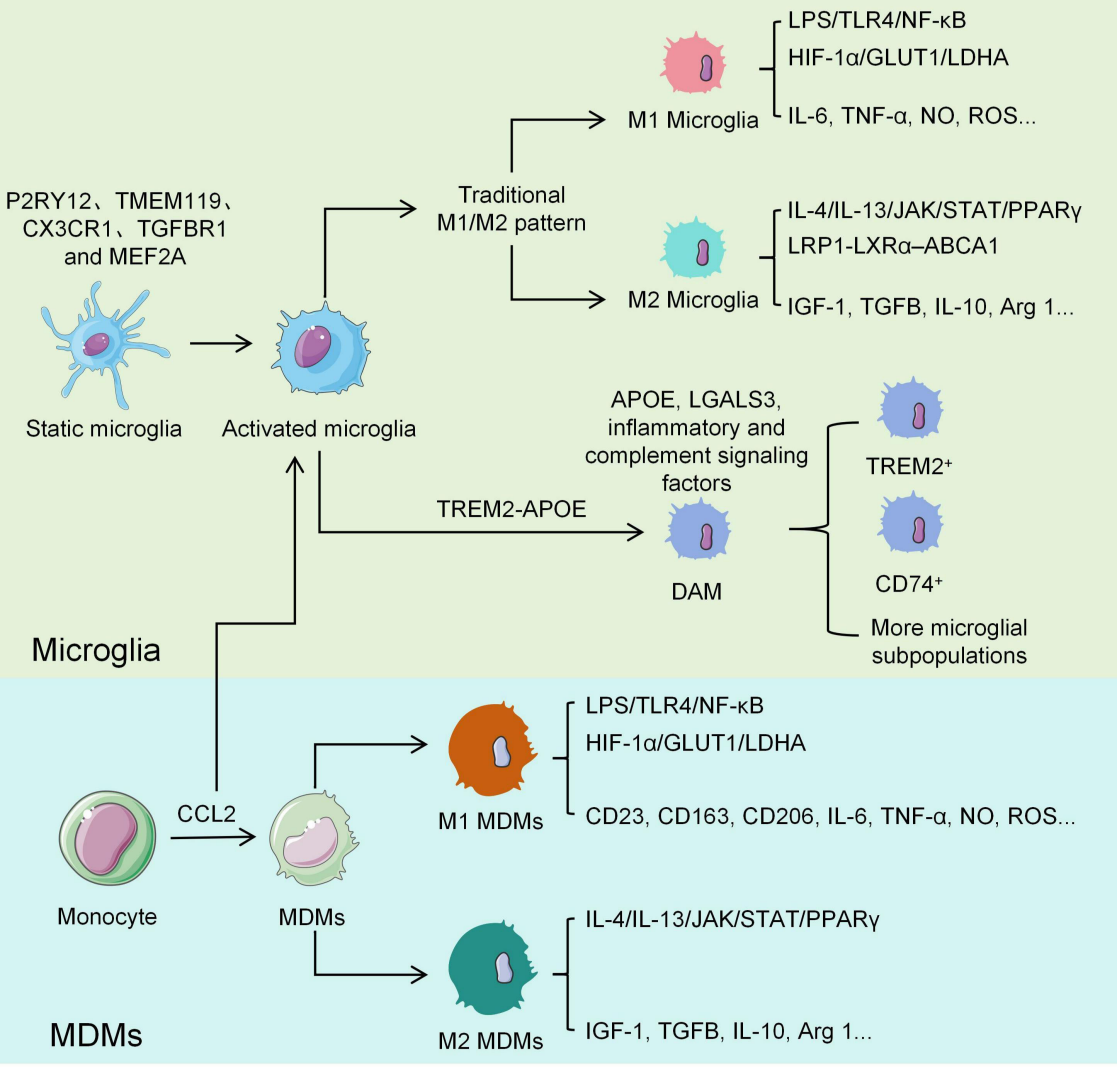
Polarization lineages and regulatory mechanisms of retinal microglia and MDMs in glaucoma. The traditional M1/M2 dichotomy fails to capture the high *in vivo* heterogeneity and dynamic plasticity of these cells; current research increasingly views their activation as a context-dependent continuum. During chronic neuroinflammation, steady-state microglia (expressing P2RY12, TMEM119 and CX3CR1, among others) progressively transition to disease-associated phenotypes (DAM/MGnD). This process is often driven by the TREM2-APOE axis and accompanied by the upregulation of genes related to complement, phagocytosis, and lipid metabolism. The early stages aid in clearing RGCs debris and limiting damage, while the advanced stages may promote synaptic pruning and neurotoxicity due to the sustained release of IL-1β, TNF-α, and ROS. MDMs rapidly infiltrate after being recruited via the BRB and CCL2-CCR2 axis. They have a stronger pro-inflammatory response during the acute phase. Subsequently, they upregulate CD163, CD206, and others to participate in tissue repair and matrix remodeling in response to repair signals. In contrast, the anti-inflammatory differentiation of microglia is more restricted. Both cell types share pro-inflammatory polarization pathways mediated by TLR4-NF-κB and HIF-1α, as well as anti-inflammatory polarization pathways mediated by IL-4/IL-13–JAK/STAT–PPARγ. However, they have distinct metabolic adaptations. Overall, both exhibit M1-biased glycolysis and M2-biased oxidative phosphorylation/fatty acid oxidation. In glaucoma, however, microglia exhibit more pronounced lipid droplet accumulation and impaired lipid clearance via LRP1-LXRα-ABCA1 (and LRP1-PPARγ) pathways. This affects their polarization trajectory and functional output.

In glaucomatous optic neuropathy, activated microglia and infiltrating MDMs do not adopt a single functional state. Instead, they undergo complex polarization processes that differentiate into distinct phenotypic subsets with specialized functions (**Figure 4**).

### 4.1 Beyond the M1/M2 dichotomy: the activation spectrum of microglia and MDMs

Traditionally, the functional polarization of macrophages has been conceptualized through the “M1/M2” dichotomous framework. This model distinguishes between a classically activated (M1) pro-inflammatory phenotype induced by interferon-γ (IFN-γ) or lipopolysaccharide (LPS) and an alternatively activated (M2) anti-inflammatory phenotype induced by interleukin-4 (IL-4) or IL-13 (88). However, the advent of single-cell sequencing and spatial transcriptomics has rendered this oversimplified model no longer adequate for accurately depicting the profound heterogeneity and dynamic plasticity of microglia and MDMs in complex *in vivo* environments. This particularly holds for chronic neurodegenerative diseases such as glaucoma (89). The current consensus is that activation states of these cells should be considered as a complex, continuous, and multidimensional spectrum (90).

Specifically, microglial activation is no longer considered a simplistic M1/M2 switch but rather a highly context-dependent continuum (91). In a healthy retinal microenvironment, microglia are maintained in a “homeostatic” state that is characterized by the expression of signature genes such as P2RY12, TMEM119, CX3CR1, TGFBR1, and MEF2A. During the chronic neural injury process of glaucoma, homeostatic microglia undergo a gradual phenotypic transition toward “disease-associated microglia” (DAM) or a “microglial neurodegenerative” (MGnD) phenotype (92). The evolution of the DAM phenotype typically depends on activation of the TREM2-APOE signaling pathway. This process is characterized by the downregulation of homeostatic genes, along with the significant upregulation of genes related to phagocytosis and lipid metabolism, including APOE, LGALS3, inflammatory cytokines, and complement signaling components (92, 93). During early glaucoma, the DAM phenotype primarily exerts neuroprotective effects by phagocytosing dead RGCs debris and isolating the injury. However, microglia persistently arrested in this state may excessively release interleukin-1β (IL-1β), tumor necrosis factor-α (TNF-α), and reactive oxygen species (ROS) during disease progression, which triggers excessive synaptic pruning and a neurotoxic cascade (89). Single-cell transcriptomic analyses have further elucidated the heterogeneity of microglia. For instance, studies of the human glaucomatous retina have identified a distinct DAM population characterized by significantly elevated expression of TREM2 and other neurodegeneration-associated genes (94). Additionally, early and dynamic changes in a CD74^+^ microglial population have been detected within the distal optic nerve, suggesting that these profiles may serve as key hallmarks of ocular hypertension-induced neurodegeneration (95).

Microglia and MDMs function synergistically within the injured microenvironment, but their activation profiles differ significantly because of their distinct developmental origins and tissue adaptations. Peripherally infiltrating MDMs have a polarization pattern that aligns closely with the classical macrophage activation paradigm (96). Following BRB disruption or recruitment of chemokines such as CCL2 into the retina and optic nerve head (97), MDMs often mount a more rapid and robust response. They tend to polarize into a state that potently secretes pro-inflammatory cytokines during the acute injury phase, which rapidly amplifies local inflammation (98). Conversely, MDMs broadly upregulate markers such as CD23, CD163, and CD206 upon induction by microenvironment repair signals and adopt a relatively comprehensive anti-inflammatory and tissue repair phenotype (99). Meanwhile, the anti-inflammatory differentiation of microglia is comparatively restricted and typically expresses only a few selected molecules such as CD209 (98).

Regarding spatiotemporal responses and functional localization, microglia are extremely sensitive to early microenvironmental stress (such as mild IOP fluctuations). Their transition to the DAM state spans the entire chronic course of glaucoma and plays a predominant role in the phagocytosis of myelin debris and synaptic remodeling (85, 100). Conversely, MDMs respond more to high-intensity signals within the local microenvironment (such as extensive cell death). Their activation not only exacerbates early neuroinflammation, but also plays an indispensable role in extracellular matrix remodeling and fibrotic repair at the optic nerve head during the later stages of the disease (101, 102).

### 4.2 Polarization-driving signaling networks: chemokine axes and molecular pathways

Within the retinal microenvironment, chemokine networks orchestrate the recruitment of MDMs and activation and polarization of microglia. The CCL2-CCR2 chemokine axis plays a pivotal role (103). In experimental glaucoma models, elevated IOP or RGC injury rapidly induces CCL2 expression and release by retinal Müller cells, microglia, and vascular endothelial cells (9, 104). This chemokine has dual regulatory functions (106). On one hand, CCL2 acts as a potent chemoattractant: it guides MDMs to cross the compromised BRB and infiltrate the retina and optic nerve head by binding CCR2 on the surface of MDMs during the early phase of disease (within hours to days). On the other hand, CCL2 can activate CCR2-expressing microglia, which promotes their migration toward lesion sites and induces morphological transformation. This amplifies inflammatory cascades (9). Consequently, the CCL2-CCR2 axis not only temporally links acute stress to chronic inflammation but also spatially couples the vascular compartment with the retinal parenchymal microenvironment (14).

The key signaling pathways that regulate polarization are highly conserved across both cell types. M1 polarization primarily depends on the activation of the Toll-like receptor 4 (TLR4)–nuclear factor κB (NF-κB) pathway: LPS binding to TLR4 triggers NF-κB nuclear translocation and upregulates the transcription of M1-associated inflammatory genes (107, 108). Concurrently, hypoxia-inducible factor-1α (HIF-1α) plays a key regulatory role in M1 polarization by upregulating glycolysis-related genes, including glucose transporter 1 (GLUT1) and lactate dehydrogenase A (LDHA). These supply the metabolic support required for this pro-inflammatory phenotype (109, 110). In contrast, M2 polarization relies largely on the activation of the peroxisome proliferator-activated receptor gamma (PPARγ) pathway (111). Signaling through IL-4 and IL-13 activates the Janus kinase/signal transducer and activator of transcription (JAK/STAT) cascade (which upregulates PPARγ expression) and promotes the secretion of anti-inflammatory factors and activation of phagocytosis-related genes (112).

Notable differences exist despite these similarities. Research indicates that microglia adopt a specific polarization pathway in glaucomatous eyes: the low-density lipoprotein receptor-related protein 1 (LRP1)–liver X receptor alpha (LXRα)–ATP-binding cassette transporter A1 (ABCA1) signaling axis. In acute glaucoma models, aberrant lipid droplet accumulation within microglia, combined with downregulated LRP1 expression, leads to insufficient PPARγ activation and subsequent impairment of lipid clearance. This disrupts M2 polarization (113). Both cell types secrete IL-10, but their secretion levels and regulatory mechanisms differ (85). Under M1-polarized conditions, microglia have high levels of IL-10 expression than MDMs. Conversely, MDMs require lipopolysaccharide (LPS) stimulation during M2 polarization to induce IL-10 expression, whereas microglia show no such dependence, which directly contributes to distinct functional plasticity under identical stimuli.

### 4.3 Metabolic reprogramming in polarization

Metabolic reprogramming is a pivotal mechanism underlying the polarization of two distinct cellular phenotypes. These phenotypes share a common metabolic dichotomy: the M1 state is predominantly glycolytic, whereas the M2 state relies on oxidative phosphorylation (OXPHOS) and fatty acid oxidation (FAO) (57). For instance, the upregulation of glycolytic pathway components, such as Glut1, hexokinase 2, and lactate dehydrogenase, enhances glucose uptake and lactate production in macrophages, which promotes M1 polarization and provides an energetic and metabolic basis for the synthesis of pro-inflammatory factors and ROS generation (109). In contrast, M2 polarization primarily relies on OXPHOS and FAO, which enhance mitochondrial function and improve ATP production efficiency to meet the energy demands of processes including phagocytosis and tissue repair (114, 115).

Under glaucomatous conditions, the metabolic profiles of these two cell types diverge considerably. In glaucoma models, impaired lipid clearance in microglia drives significant lipid droplet accumulation, which is a key factor in promoting their M1 polarization. Conversely, activation of the LRP1-PPARγ pathway enhances cholesterol efflux, reverses lipid droplet accumulation, and induces a shift toward M2 polarization (113). Under equivalent oxidized low-density lipoprotein (ox-LDL) stimulation, MDMs demonstrate greater lipid uptake capacity and higher expression of pro-inflammatory cytokines than microglia; ox-LDL preferentially elevating fatty acid metabolites in MDMs, while primarily increasing phospholipid metabolites in microglia (116). Additionally, microglial mitochondrial function appears to be better adapted to high oxygen consumption in the retina (117). Studies have indicated that promoting autophagy to eliminate damaged mitochondria drives microglial polarization toward the anti-inflammatory M2 phenotype, helping to maintain metabolic homeostasis and attenuate pro-inflammatory signaling (118). These findings highlight metabolic modulation as a promising therapeutic strategy for regulating microglial function in glaucoma.

In summary, the intrinsic differences in the transcriptional regulatory networks and energy metabolism profiles between microglia and MDMs fundamentally shape their distinct roles in the pathological progression of glaucoma from early neuroprotection to late-stage neurodegeneration. Elucidating these disparities not only deepens the understanding of the immunopathological mechanisms underlying glaucoma but also provides a solid theoretical foundation for the future development of precision therapeutic strategies targeting specific metabolic targets (such as the LRP1-PPARγ axis) or chemokine cascades (such as the CCL2-CCR2 axis).

## 5 Activation and polarization of microglia and MDMs in glaucoma

**Figure 5:**
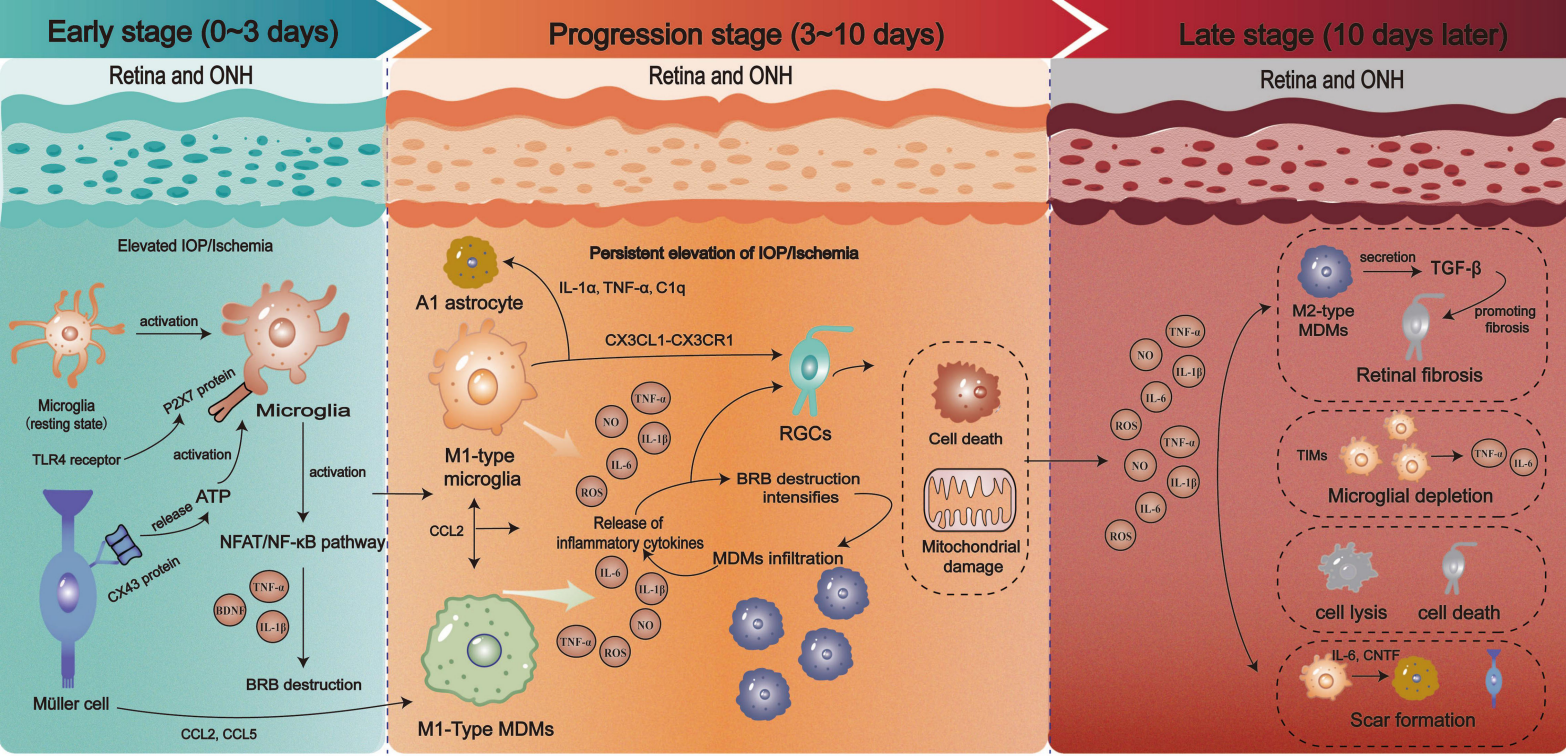
Activation and polarization of microglia and MDMs in glaucoma. Microglia rapidly shift from a branched resting form to amoeboid morphology during the early stage and upregulate TLR4 and P2X7R, perform debris clearance, and release low-level TNF−α, IL−1β, and BDNF. Müller cell–derived ATP amplifies activation and promotes limited macrophage entry via increased BRB permeability. Microglia polarize toward M1 and produce TNF−α, IL−6, NO, and ROS. This drives RGC death and BRB breakdown. Macrophage infiltration intensifies, sustaining inflammation despite partial M2 induction. During the late stage, microglia show “disease-associated” dysfunction with reduced phagocytosis and persistent oxidative stress, while macrophages shift toward profibrotic phenotypes, contributing to chronic injury and structural remodeling.

Glaucoma is characterized by the progressive degeneration and loss of RGCs and is accompanied by optic nerve damage. Neuroinflammation plays a pivotal role in disease progression and is driven by “dual immune armies” comprising microglia and MDMs. The activation states, polarization phenotypes, and intercellular interactions of these immune cells evolve dynamically throughout the disease course. Therefore, a detailed stage-specific dissection of intercellular communication networks is essential for elucidating how immune responses regulate neuronal survival and degeneration (**Figure 5**).

### 5.1 Early stage: immune surveillance and protective activation (approximately 0–3 days)

During the early stages of glaucoma, elevated IOP or other initial injury signals activate the microglia in the retina and optic nerve head. Studies have demonstrated that microglia undergo a morphological transition from a quiescent dendritic state to an activated phenotype for 4 h starting from 24 h after IOP increase (119). Their cell bodies undergo retraction with diminished processes and a rapidly responsive amoeboid morphology, which are the hallmarks of an activated state (120). In terms of the temporal sequence of optic nerve injury, microglia initiate their response within 24 h of injury and are fully activated 2–3 days afterward (16). At this stage, microglia upregulate pattern recognition receptors (such as TLR4) and ion channels (such as P2X7R) while primarily retaining immune surveillance capabilities (119, 121). They achieve this by phagocytosing cellular debris and secreting low levels of pro-inflammatory mediators (such as TNF-β) and neurotrophic factors (such as BDNF). This clears injury-derived products and maintains microenvironmental homeostasis (8).

Concurrently, a complex communication network emerges among multiple glial cell types to enable the collective sensing and amplification of the initial insult. Notably, Müller cell activation may occur before microglia (122). Activated Müller cells release ATP via connexin 43 hemichannels, which activate P2X7 receptors on microglial membranes and trigger the calcium-dependent NFAT/NF-κB signaling pathway, a key initiator of microglial activation (123). This interglial crosstalk further amplifies the initial injury signal. Concurrently, chemokines such as CCL2 and CCL5 released by both microglia and Müller cells enhance BRB permeability and facilitate the infiltration of MDMs into the injured retina (124). At this stage, MDMs are relatively scarce and may demonstrate an incompletely polarized phenotype. Microglia establish bidirectional communication with RGCs via the CX3CL1-CX3CR1 axis, while neuron-derived CX3CL1 continuously suppresses microglial overactivation to maintain the homeostatic phenotype (125). During the initial phase of IOP elevation (12–24 h), transient upregulation of CX3CL1 provides neuroprotective negative feedback. However, CX3CL1 expression declines if the injury is sustained beyond 48 h, which increases the suppression of microglia and marks an early molecular shift toward neurotoxicity (126). Additionally, microglia recognize vulnerable RGC synapses via complement C1q, which initiates selective synaptic pruning. This process that aids in neuroprotective circuit remodeling, but prolonged activation leads to pathological synaptic engulfment (47).

Moreover, the early immune response in glaucoma has systemic features. Reactivation of quiescent microglia is observed not only in the injured eye but also in the contralateral eye within 24 h of unilateral laser-induced ocular hypertension (127). This observation suggests that systemic immune mechanisms contribute to the rapid activation of microglia in the uninjured retina.

### 5.2 Progression stage: neurotoxic polarization and inflammatory amplification (approximately 3–10 days)

When the IOP remains elevated or ischemic injury persists, neuroinflammation progresses to the amplification phase. Sustained injury signals prompt microglia to undergo classical activation (M1 polarization (128). M1-polarized microglia have a highly pro-inflammatory phenotype, secreting substantial amounts of neurotoxic substances such as TNF-α, IL-1β, IL-6, NO, and ROS (6). These factors not only directly induce RGC apoptosis and mitochondrial dysfunction but also further compromise the BRB. This facilitates increased MDM infiltration and forms an inflammatory positive feedback loop (13). Moreover, IL-1α, TNF-α, and C1q secreted by early-activated microglia induce the transformation of astrocytes into a neurotoxic (A1) phenotype, stripping them of their neurotrophic functions and prompting the release of complement components that exacerbate neuroinflammation (129).

BRB disruption intensifies with MDMs beginning to infiltrate the core lesion area in large numbers approximately 3 days post-injury and reaches peak infiltration by 1 week post-injury (16). Retinal perivascular macrophages facilitate the traversal of MDMs across the BRB via expression of MHC class II molecules (130). Multiomics analyses have confirmed that the infiltration levels of M1 macrophages and CD4+ T cells correlate with disease severity in patients with primary open-angle glaucoma (131). Local microenvironmental factors such as TGF−β and IL−4 promote polarization of a subset of MDMs toward the M2 phenotype, which expresses Arg1 and CD206 to exert anti−inflammatory and pro−repair effects. However, this shift exerts limited influence during the progressive stage (132).

At this stage, MDMs and microglia engage in bidirectional communication through direct contact or soluble factors, mutually modulating their respective functions (133). MDMs exacerbate the local pro-inflammatory milieu and reinforce microglial M1 polarization. Conversely, persistently activated M1 microglia continuously release chemokines to sustain MDM recruitment (16). Overall, the M1 proinflammatory phenotype becomes dominant, disrupting the M1/M2 dynamic equilibrium and establishing a self-perpetuating, vicious inflammatory cycle (134). Abnormal activation of the complement system synergistically enhances early inflammatory cascades in retinal ischemia/reperfusion (I/R) by promoting microglial antigen presentation, MDM infiltration, and BRB disruption. This ultimately aggravates neural injury (135).

### 5.3 Late stage: chronic inflammation and repair failure (beyond 10 days)

In chronic or late-stage glaucoma, neurodegeneration may progress despite controlled IOP (136). At this stage, a significant RGC loss has occurs, resulting in optic nerve atrophy. The “dual immune armies” enter a functionally exhausted state, transitioning from acute pro-inflammatory toxicity to repair failure and chronic inflammation.

Long-term excessive activation leads to exhaustion-like changes in microglia that are characterized by diminished phagocytic capacity yet persistent inflammatory secretion (137). This may be accompanied by the dysregulation of inflammatory and neuroprotective gene expression, mitochondrial dysfunction, and exacerbated oxidative stress (138). Although their phenotype remains M1-biased, their TNF-α secretion capacity diminishes, shifting instead to extensive release of ROS, lipid peroxidation products, and pro-fibrotic factors (139). This perpetuates chronic injury states, further damaging surviving ganglion cells. Transcriptomic and chromatin accessibility analyses in a study revealed a population of activated microglia (termed terminally inflamed microglia, TIMs) co-expressing inflammatory signaling and intrinsic cellular stress markers, the abundance of which increased with age and APOE4 load (140). Therefore, this study proposes that TIMs represent an “exhaustion-like state” of microglia that contributes to increased disease risk and pathological progression in APOE4 carriers and older adults. This suggests their potential as therapeutic targets for glaucoma.

Persistent low-level infiltration of MDMs continues into the later stages, although their responsiveness to injury gradually declines over time (51). As the disease progression, the reparative M2 polarization response is often attenuated, whereas fibrosis-related signaling intensifies, potentially promoting a pro-fibrotic MDM phenotype (141). Such MDMs contribute to retinal tissue fibrosis by secreting fibrotic mediators such as TGF-β and acting in concert with circulating fibroblasts, thereby further disrupting normal retinal architecture (142).

During the later stages, interactions among microglia, astrocytes, and Müller cells culminate in the formation of dense glial scars (16). Reactive astrocytes and Müller cells undergo hypertrophy and proliferation, and their processes interweave to encapsulate damaged areas. While this physical barrier helps sequester inflammation and limits the spread of damage, it also severely impedes axonal regeneration and extension. Dysfunctional microglia continually stimulate astrocyte reactivity by releasing factors such as IL-6 and ciliary neurotrophic factor (CNTF), which maintains the scar structure. This aberrant microglia-glial-RGC crosstalk ultimately strips the surviving neurons of their regenerative capacity, shifting the microenvironment from promoting survival to actively inhibiting repair.

### 5.4 Critical junctures in neuroprotection and neurotoxicity conversion

Synthesizing the characteristics of the aforementioned stages, the transition from the early to the progressive phase represents a critical time window for the functional shift of the “dual immune armies” from neuroprotection to neurotoxicity, particularly between 72 h and one week after sustained IOP elevation (143). Key molecular triggers likely involve purine signaling imbalance, complement cascade activation, and establishment of a microglia-astrocyte toxicity circuit. For example, sustained ATP release and accumulation of its metabolite, adenosine, through synergistic action on P2X7 and adenosine receptors drive microglia toward a pro-inflammatory phenotype (144). Furthermore, deposition of complement proteins such as C1q and C3 at RGCs synapses not only mediates excessive pruning of vulnerable synapses by microglia but may also amplify their pro-inflammatory program by activating complement receptors on microglia (145). Besides, induce astrocytes to adopt the A1 phenotype released by microglia reach a certain threshold (129). These acquired toxic astrocytes lose their neurotrophic functions and amplify neurotoxicity in concert with microglia. This may mark a transition from a reversible protective response to an irreversible damage cascade.

At different pathological stages of glaucoma, the behavioral patterns of the “dual immune armies” undergo profound evolution: they progress from rapid immune surveillance and protective responses to a neurotoxic positive feedback loop dominated by M1-type polarization and ultimately enter a late-stage state characterized by chronic inflammation and impaired repair (146). There is a growing body of evidence highlighting proresolving mediators, particularly lipoxins, as promising therapeutic candidates for glaucoma. Notably, lipoxin B4 (LXB4) maintains a homeostatic microglial phenotype in the optic nerve while suppressing proinflammatory activation associated with disease, representing a potential neuroprotective mechanism that targets microglia (95). Enhancing the lipoxin pathway is also a promising strategy for counteracting reactive astrogliosis in neurodegenerative disorders (147). Lipoxins have been shown to markedly reduce Müller glial reactivity following glaucomatous optic nerve injury (148). Interestingly, Müller glia are a major endogenous source of LXB4 in the retina, and this neuroprotective Müller glial–lipoxin axis is amplified during chronic transient receptor potential vanilloid 4 (TRPV4) activation (149). Therefore, effective control of neuroinflammation during the early or progressive stages of glaucoma by blocking the abnormal activation of microglia, excessive MDMs infiltration and dysregulated gliosis can theoretically interrupt the vicious cycle of inflammation-induced damage and halt or delay further progression of glaucomatous optic nerve damage. Therefore, the sequential modulation of the immune microenvironment at different disease stages holds promise as a key therapeutic strategy for achieving effective optic nerve protection.

## 6 Targeted regulation of microglia and MDMs for glaucomatous optic nerve protection

The onset and progression of glaucomatous optic neurodegeneration are closely linked to an immune imbalance mediated by microglia and MDMs, which play distinct roles across disease stages. As resident immune sentinels in the retina, appropriately timed and moderately early microglial activation can be neuroprotective, whereas excessive activation and maladaptive polarization contribute to RGC injury. During disease progression, the large-scale infiltration of MDMs and their pro-inflammatory polarization exacerbate neuronal damage. Therefore, moving beyond conventional broad-spectrum immunosuppression toward cell type–specific and stage-tailored immunomodulatory strategies represents a key direction for neuroprotective therapies in glaucoma.

### 6.1 Regulation of cell development and recruitment

Interfering with the origin and trafficking of microglia and MDMs is the primary approach for achieving cell-type-selective regulation. The colony-stimulating factor 1 receptor (CSF1R) is a pivotal regulator of microglial survival and proliferation (150). Short-term CSF1R inhibition during the acute phase attenuate neurodegeneration and delays disease progression (151). In RI/R models, intravitreal administration of a CSF1R-neutralizing antibody effectively prevents excessive microglial activation, suppresses T cell recruitment, markedly improves RGC survival, and preserves visual function (152). However, long-term and complete microglial depletion aggravates neuronal injury in glaucoma models (153), indicating a strict time window dependency. This strategy may be better suited for short-term intervention (for example, 2–4 weeks) after acute IOP spikes rather than for the long-term management of chronic disease. Future studies should focus on developing reversible or stage-specific CSF1R modulators or exploring combination regimens (such as with CX3CR1 agonists) to suppress pathological activation while preserving microglial homeostatic functions.

In contrast with CSF1R-based approaches, blocking the CCR2–CCL2 chemokine axis enables selective control of MDM recruitment without directly affecting microglia. In chronic ocular hypertension models, CCL2 knockout significantly reduces retinal MDM density and mitigates RGC damage, and this protection occurs without changes in the expression of pro-inflammatory factors (C1q, IL-1α, TNF-α) (106). These findings suggest that the CCL2 blockade primarily limits MDM infiltration while preserving the innate immune function of microglia, thereby reducing the risk of infection associated with broad immunosuppression. This mechanism also implies that CCL2 expression levels in the aqueous humor or serum may serve as liquid biopsy indicators that reflect the extent of MDM infiltration. Nonetheless, the complete blockade of MDM recruitment may impair tissue repair functions (such as debris clearance). Therefore, future interventions should explore partial inhibition rather than full ablation.

Lipocalin 2 (LCN2) promotes MDM infiltration by disrupting the blood–retinal barrier (BRB) and downregulating tight junction proteins (ZO-1, occludin, and claudin-5) (154). In acute ocular hypertension models, RNA sequencing identified Lcn2 as one of the most strongly upregulated inflammation-related genes (135). Lcn2 knockdown substantially alleviates BRB disruption, suppresses ICAM-1 and CCL2 expression, and reduces MDMs infiltration. Thus, LCN2 may serve as a biomarker of neuroinflammation severity in acute glaucoma and as an early therapeutic target. However, complete LCN2 inhibition may cause adverse effects due to its physiological role in antimicrobial immunity and iron metabolism and requires careful safety evaluation.

### 6.2 Targeted cell activation and polarization

After recruitment, the regulation of the activation states and polarization trajectories becomes central to precise immunomodulation. The neuron-derived chemokine CX3CL1 and its specific receptor CX3CR1 form a signaling axis critical for maintaining microglial quiescence (126). CX3CR1-deficient mice have exaggerated neurotoxic responses and widespread RGC loss (75). Minocycline-and Müller cell–derived extracellular vesicles delivering miR-125b-5p and miR-16-5p consistently suppress excessive microglial activation by positively modulating this axis (125). Therefore, pharmacological targeting of CX3CR1 has the potential to achieve precise modulation of microglial function through immune response reprogramming.

The cGAS–STING pathway is a key molecular driver of retinal neuroinflammation in glaucoma; its overactivation promotes M1-like polarization of microglia and aggravates neuronal injury (155). The A151 oligonucleotide competitively inhibits cGAS and AIM2 activities, promoting a shift from M1-like toward M2-like phenotypes and exerting neuroprotective effects (156). Targeting the downstream components of this pathway, such as TBK1 inhibitors or antibodies against the type I interferon receptor, has also been shown to improve RGC survival (157). cGAS–STING is central to broad antiviral immunity, and systemic inhibition may increase the susceptibility to infection. Local intraocular delivery (such as via intravitreal injection) can limit systemic exposure and reduce off-target effects but requires optimization of sustained-release performance and the safety of repeated administration.

### 6.3 Modulation of cellular setabolic state

The metabolic state is a fundamental determinant of polarization phenotypes and effector functions in microglia and MDMs. Therefore, metabolic reprogramming has emerged as a frontier in precise immunomodulation of glaucoma (158). Clinical studies have shown that LRP1 expression is significantly reduced in the serum of patients with glaucoma and in the retinas of model animals, accompanied by abnormal lipid droplet accumulation and enhanced M1-like polarization (113). Nanoparticles loaded with alpinetin (AlpNPs) activate the PPARγ–LXRα–ABCA1 pathway, promote cholesterol efflux, restore lipid metabolic homeostasis, and induce M2-like polarization with neuroprotective effects (113). Additionally, STK11 deficiency can integrate IL-4 signaling and enhance glutamine metabolism, which further promotes M2-like polarization (159). Accordingly, targeting STK11 and related amino acid metabolic pathways may offer more fine-grained and precise metabolic intervention for optic nerve protection in glaucoma and expand the repertoire of immunomodulatory strategies.

### 6.4 Barriers to clinical translation and future perspectives

The strategies above show promise in experimental models. However, barriers remain for translating neuroprotective immunomodulation into clinical glaucoma therapy. First, there are discrepancies between models and human diseases. Many existing animal models primarily mimic acute injury, whereas primary open-angle glaucoma in humans is a chronic, progressive disorder. Early immunointerventions validated in acute models may fail or even become detrimental during chronic diseases because therapeutic windows are difficult to define. Second, there is a dual nature of the targets and insufficient intervention precision. Both microglia and MDMs can be neuroprotective or neurotoxic depending on the context. Current interventions struggle to distinguish and control these functional states selectively. A major challenge in drug design is preserving the trophic support provided by microglia, while precisely suppressing the toxic effects of MDMs. Third, there are limitations to drug delivery systems. Intravitreal injection bypasses the BRB, but it is invasive and unsuitable for the long-term treatment of chronic diseases. In contrast, systemic administration is associated with risks of off-target effects and immunosuppression. A key way to overcome this challenge is to develop BRB-penetrant cell-selective delivery platforms (such as AlpNPs). Finally, biomarkers are lacking. Clinical translation requires biomarkers that can monitor the treatment response and guide patient dosage. Currently, non-invasive real-time methods for assessing retinal neuroinflammation are limited. While molecules such as CCL2 and LCN2 in the aqueous humor show promise as liquid biopsy biomarkers, large clinical cohorts are needed to validate their sensitivity and specificity.

In summary, targeted regulation of the development, recruitment, activation, polarization, and metabolic programs of microglia and MDMs provides multiple candidate strategies for precise immunomodulatory therapy in glaucoma and highlights several biomarkers with translational potential. However, substantial challenges remain, including interspecific differences, drug-related adverse effects, limited intervention precision, and delivery system constraints. Future studies should deepen our mechanistic understanding, conduct large-scale clinical trials to advance glaucoma immunomodulatory therapies from the bench to bedside, and provide new therapeutic options for patients.

This review systematically summarizes the roles and regulatory mechanisms of microglia and MDMs—two core immune cell populations—in glaucoma. We highlight that they exhibit significant heterogeneity in their origins, functional properties, molecular markers, and response patterns to pathological stimuli although both belong to the retinal immune cell community. Optic nerve damage in glaucoma is not solely driven by elevated IOP; dysregulation of the neuroimmune microenvironment plays a pivotal role in disease progression. Through synergistic interactions and functional division of labor, microglia and MDMs cooperatively orchestrate neuroinflammatory responses. The balance of their functional states is directly correlated with RGC survival and overall disease prognosis.

The precise identification and targeted regulation of these two immune cell types opens up a novel therapeutic avenue for glaucoma that extends beyond conventional IOP-lowering strategies. Modulating the immune microenvironment is not intended to replace existing hypotensive treatments but rather serve as a crucial adjunctive therapy to address the clinical dilemma wherein optic nerve degeneration progresses despite well-controlled IOP. By maintaining microglial homeostasis, blocking aberrant MDM infiltration, or reprogramming immune cell phenotypes, neuroprotective effects can be superimposed on IOP reduction, achieving a “hypotensive-immunomodulatory” dual-target intervention. This strategy holds immense promise for delaying disease progression, preserving residual visual function, and providing a robust theoretical foundation for the development of clinically translatable interventions.
